# A formation-response classifier for borehole deviation using conventional well log data

**DOI:** 10.1371/journal.pone.0356220

**Published:** 2026-10-09

**Authors:** Naveen Kumar B, Aslam Abdullah Mohammed

**Affiliations:** School of Chemical Engineering, Vellore Institute of Technology, Vellore, Tamil Nadu, India; Henan Polytechnic University, CHINA

## Abstract

Accurate and timely identification of borehole deviation is essential in drilling, as unexpected trajectory shifts serve as key indicators of localised geomechanical failure, formation anisotropy, and wellbore integrity degradation. This study presents a systematic machine learning (ML) framework for classifying borehole deviation as Normal or deviated, utilising seven petrophysical and geomechanical well-log features from a 301-sample single-well dataset. Five supervised classifiers, K-Nearest Neighbours (KNN), Logistic Regression (LR), Gaussian Naive Bayes (GNB), Multilayer Perceptron (MLP), and Histogram Gradient Boosting (HGB) were evaluated. Preprocessing incorporated standard scaling, median imputation, and class imbalance mitigation using balanced class weights and the Synthetic Minority Oversampling Technique (SMOTE). To eliminate spatial data leakage, models were evaluated within a 10-fold depth-blocked cross-validation framework across five random seeds, yielding 95% confidence intervals for all performance metrics. The HGB model achieved the highest macro-averaged F1-score of 97.88% (±2.05% CI) and an out-of-fold cross-validation accuracy of 98.00%, outperforming KNN (94.39%), MLP (86.04%), GNB (81.04%), and LR (77.03%). Permutation-based feature importance highlighted Delta T, Gamma-ray, and Resistivity as the primary indicators of deviation risk, aligning with geomechanical principles. These findings demonstrate that Histogram Gradient Boosting with leakage-safe preprocessing provides a robust offline proof-of-concept for assessing wellbore trajectory quality before field validation.

## 1. Introduction

Borehole deviation, the unintended departure of a drill bit’s trajectory from the planned well path, represents a critical manifestation of localised formation failure and in-situ stress anisotropy. Understanding the geological and geomechanical triggers behind these deviations is essential for modelling subsurface stability, assessing caprock sealing performance, and ensuring long-term wellbore integrity in deep geological systems. As the industry transitions toward increasingly complex well geometries, extended-reach drilling (ERD), multilateral wells, and ultra-deepwater completions, the capacity to detect, predict, and prevent unintended deviation has become correspondingly more critical [1,2]. Conventional deviation monitoring is based on directed surveys taken at specific depth intervals and measurement-while-drilling (MWD) survey stations. Despite their effectiveness, these methods are, by their very nature, retrospective. Because deviation is historically discovered retrospectively, identifying these intervals provides a valuable footprint of how specific rock units physically respond to mechanical excavation and in-situ stress fields under localised conditions. The real-time subsurface data stream has been significantly enhanced by the development of logging-while-drilling (LWD) technology; however, it remains challenging to convert the abundance of petrophysical and geomechanical information into reliable indicators for detecting existing deviation [3].

Accurate geological logging and petrophysical well log analysis directly govern subsequent drilling efficiency, stimulation design, and long-term production performance. Unintended wellbore deviations distort localized bottom-hole pressure and temperature profiles, which are critical when evaluating the design and optimisation of bottom-hole temperature–pressure combinations in gas production from gas hydrates via $CO_{\text{2}}$ replacement strategies [4]. Similarly, reliable wellbore stability and geomechanical models are essential for understanding multivariate coupling behaviour and reservoir characteristics in enhanced geothermal reservoirs [5]. Furthermore, trajectory control is vital for mitigating near-wellbore tortuosity that adversely impacts the flow dynamics, sedimentation behaviour, and transport efficiency of solid proppant particles in $CO_{\text{2}}$ fracturing fluids [6].

A compelling paradigm shift is provided by machine learning (ML), supervised classifiers may be able to identify high-risk intervals in real time by learning statistical relationships between well log signatures and known deviation outcomes from historical datasets, the development of classification models that may support proactive drilling decisions when integrated with real-time LWD systems. In the present study, model development and validation were performed offline using historical well-log data [7,8]. Prior work has demonstrated the utility of artificial neural networks (ANNs) [9,10], support vector machines (SVM), and random forests (RF) [11,12] for a variety of well-log-derived activities, such as wellbore stability evaluation, pore pressure prediction, and lithology classification. Nevertheless, there are still few thorough comparison analyses in the literature on deviation classification that concurrently address class imbalance, limited sample sizes, hyperparameter optimisation, and statistical stability across several random seeds.

By providing a thorough, repeatable machine learning framework created especially for binary borehole deviation classification, our study fills this gap. The following are the contributions made by this work:

- Five algorithmically different supervised classifiers (KNN, LR, GNB, MLP, and HGB) were systematically compared under the same preprocessing and assessment settings.
- A leakage-safe dual-strategy approach to class imbalance combining cost-sensitive balanced class weighting and Synthetic Minority Oversampling Technique (SMOTE), where SMOTE and scaling are strictly confined within training folds via an imbalanced pipeline.
- Multi-seed(n = 5) depth-blocked sequential cross-validation (K-fold without shuffling on depth-sorted data), providing a strict spatial barrier between folds and yielding 95% Student t-distribution confidence intervals across all performance metrics.
- Pipeline-safe permutation feature importance analysis on the optimal Histogram Gradient Boosting model, linking algorithmic feature rankings directly to geomechanical principles of formation elasticity and clay content.
- The proposed framework is intended as an offline proof-of-concept for future real-time implementation using streaming LWD measurements.

This is how the rest of the paper is organised. The relevant literature on machine learning applications in drilling and well log analysis is reviewed in Section 2. The dataset and preprocessing technique are explained in Section 3. The experimental design, including methods, hyperparameter search, and evaluation process, is described in depth in Section 4. Results are presented and discussed in Section 5. Implications and future study directions are discussed in Section 6’s conclusion.

## 2. Literature review

### 2.1 Machine learning in drilling engineering

Over the past ten years, there has been a noticeable increase in the use of data-driven approaches to drilling engineering challenges due to the growth of real-time downhole sensor data and improved computational accessibility. Early research by Elkatatny et al. [10] proved that ANNs could use surface readings to forecast drilling parameters like rate of penetration (ROP) with competitive accuracy. Later research expanded machine learning techniques to formation assessment tasks, such as lithology detection [13]. mineralogy prediction from spectroscopy logs [14], and fluid contact detection [15]. Testing accuracy is increased to 97.03% by chaining AdaBoost with Random Forest, which removes severe overfitting. Three essential reservoir characteristics are automatically extracted by the hybrid architecture: water saturation, rock porosity, and shale volume. Using conventional logs and input data such as resistivity and gamma rays, it reconstructs missing shear-wave logs with 98.54% accuracy. During drilling campaigns, this automated method offers a reliable, low-risk workflow for real-time digital petrophysical interpretation [12]. The comparative study demonstrated that the optimised Random Forest (RF) model outperformed other algorithms, achieving a high testing accuracy of 96.67% and a cross-validation accuracy of 98.31% in predicting wellbore deviation [16]. More recently, deep learning architectures, including Recurrent Neural Networks(RNN) have been applied to time-series representations of drilling parameters to anticipate stuck pipe events [17], which are often causally related to severe deviation.

### 2.2 Well log feature engineering for deviation studies

The selection of input features for deviation-related ML tasks is guided by geomechanical principles. Gamma-ray (GR) logs are widely recognised as proxies for shale content and formation ductility [18] Argillaceous intervals where creep under differential load is a major mechanical source of drill trajectory modification are indicated by high GR values. Rock stiffness is directly reflected in acoustic logs, which include compressional interval transit time (ΔT) and the derived velocities V_p_ and V_s_ [19,20].with mechanically weak periods showing decreased V_p_ and increased ΔT. Complementary limitations on formation competence are provided by bulk density and density-derived porosity [21], Poisson’s ratio, which may be calculated from V_p_/V_s_, is a sensitive indicator of wellbore closure risk because it controls lateral deformation under axial load [22].

Because resistivity logs are sensitive to fluid saturation, they provide deviation-relevant information. Low resistivity intervals may indicate over-pressured or fractured zones, where formation weakening may result in bit walk [23].The inclusion of shale volume (V_sh_) as a derived feature consolidates multiple log responses into a single dimensionless index of argillaceous content widely used in petrophysical workflows [24].

### 2.3 Class imbalance in drilling data

Class imbalance is a common problem in drilling machine learning studies: in a typical vertical well dataset, intervals labelled as “normal” significantly outnumber deviated intervals. While significantly under classifying the minority (deviated) class, exactly the class of operational interest, naive classifiers trained on unbalanced data typically show inflated overall accuracy. For imbalanced binary classification, Chawla et al. [25] presented SMOTE, which creates synthetic minority samples by interpolating between existing minority neighbors and proved its superiority over basic oversampling and undersampling techniques. To stop test sample information from leaking into the synthetic generation process, later research has argued that SMOTE should only be used in training folds within cross-validation loops [26]. The alternative method, known as balanced class weighting, which is natively supported by most scikit-learn estimators, has been demonstrated to enhance SMOTE when combined with it. It imposes inversely proportionate loss penalties to the majority and minority classes [27].

### 2.4 Evaluation metrics for imbalanced classification

When class distributions are distorted, standard accuracy is a deceptive statistic. The chosen primary metric for imbalanced situations is the macro-averaged F1-score, which weights precision and recall equally across classes, independent of class frequency [28].The AUC-ROC is threshold-invariant and characterises discriminative ability across all potential classification thresholds [29] When sample counts are not comparable across classes, the Matthews Correlation Coefficient (MCC), a single scalar that captures all four cells of the confusion matrix, is very useful [30] To present a comprehensive diagnostic picture, this study reports all three parameters in addition to per-class precision, recall, and F1-score.

### 2.5 Research gap and positioning

There are several recurring gaps in the evaluated literature, as shown in Table 1. First, just a few studies include more than two or three algorithms under identical experimental conditions, which limits direct head-to-head comparability, even though ensemble techniques like Random Forest and gradient boosting routinely outperform linear and probabilistic classifiers. Second, most evaluated work lacks multi-seed stability analysis, which measures whether reported metric differences are the result of random partitioning artefacts or true model superiority. Third, when class imbalance correction is used, it is often done in a way that puts test-set data at risk of being leaked into the training pipeline (e.g., applying SMOTE before splitting). All three holes are immediately addressed by the current study.

**Table 1:** Summary of related literature on ML applications in well log analysis and drilling classification.

Author & Year	Method	Dataset	Features	Key Finding
Ahmed S. et al. [10]	Artificial Neural Network (ANN)	Surface drilling data	WOB, RPM, Torque, Flow rate, ROP	ANN accurately predicted drilling performance parameters from surface measurements.
Ren et al. [13]	Machine Learning Classification	Well log dataset	Gamma ray, Density, Resistivity, Neutron logs	ML effectively classified lithology using conventional well logs.
Bhatt & Helle [14]	Machine Learning Regression	Spectroscopy log dataset	Spectroscopy logs	Successfully predicted rock mineralogy from spectroscopy measurements.
Hill [15]	Pattern Recognition	Formation evaluation dataset	Wireline logs	Automated identification of fluid contacts from well log responses.
Srivardhan [12]	AdaBoost + Random Forest	Conventional well logs	Gamma ray, Resistivity, Density, Sonic logs	The hybrid model minimized overfitting and accurately predicted reservoir properties. Reconstructed missing shear-wave logs with high prediction accuracy.
Bhattacherjee, Ahmed & Sutradhor [16]	Optimized Random Forest	Borehole deviation dataset	Well logs and drilling parameters	RF achieved the best performance for borehole deviation prediction.
Mopuri et al. [17]	Recurrent Neural Network (RNN)	Time-series drilling data	WOB, Torque, RPM, Hook Load, Mud Flow	RNN successfully predicted stuck pipe events using sequential drilling data.
Asquith, Krygowski & Gibson [18]	Petrophysical Analysis	Wireline logs	Gamma Ray (GR)	GR is an effective indicator of shale content and formation ductility.
Vernik & Liu [19]	Rock Physics Analysis	Sonic log dataset	ΔT, V_p_	Acoustic logs provide reliable estimates of rock stiffness.
Mavko, Mukerji & Dvorkin [20]	Rock Physics Modelling	Elastic property dataset	V_p,_ V_s_, ΔT	Elastic parameters strongly correlate with the mechanical strength of the formation.
Rider & Kennedy [21]	Petrophysical Interpretation	Density log dataset	Bulk Density, Density Porosity	Density-derived parameters estimate formation competence.
Fjaer et al. [22]	Geomechanical Modeling	Rock mechanics dataset	Poisson’s Ratio, V_p_/V_s_	Poisson’s ratio is a key indicator of wellbore stability and deformation.
Zoback [23]	Geomechanical Analysis	Resistivity log dataset	Resistivity	Low resistivity often indicates fractured or over-pressure formations associated with deviation risk.
Schlumberger [24]	Petrophysical Workflow	Multi-log dataset	Shale Volume (V_sh_)	V_sh_ combines multiple log responses into a robust shale-content indicator.
Chawla et al. [25]	SMOTE	Imbalanced benchmark datasets	Minority class samples	SMOTE effectively addressed class imbalance by generating synthetic minority samples.
Blagus & Lusa [26]	SMOTE with Cross-validation	High-dimensional datasets	Training samples	It is recommended to apply SMOTE only within training folds to prevent data leakage.
He & Garcia [27]	Cost-sensitive Learning	Imbalanced classification datasets	Class weights	Balanced class weighting improved minority class recognition in imbalanced datasets.
Sokolova & Lapalme [28]	Macro F1-score Evaluation	Classification datasets	Precision, Recall	Macro-F1 provides an unbiased evaluation for imbalanced classification problems.
Fawcett [29]	ROC Analysis	Binary classification datasets	ROC Curve	AUC-ROC effectively measures classifier discrimination across thresholds.
Chicco & Jurman [30]	Matthews Correlation Coefficient (MCC)	Binary classification datasets	Confusion Matrix	MCC is one of the most reliable metrics for evaluating imbalanced binary classifiers.

## 3. Methodology

### 3.1 Data description

The study dataset consists of 301 depth-indexed well-log samples collected across a continuous depth interval of 4,000–4,300 m True Vertical Depth (TVD) from a single vertical well using wireline and industry-standard Logging-While-Drilling (LWD) instruments. The dataset serves as a benchmark for offline methodological validation rather than direct operational field control.

Following quality control and preprocessing, the Neuron Porosity curve was excluded from model inputs as neutron logging was not acquired across this borehole interval. Secondary calculated logs, including Density Calculated, Density Porosity, and Poisson’s Ratio, were omitted to eliminate mathematical redundancy and multicollinearity. Seven petrophysical and geomechanical features are retained for model training, as shown in Table 2. Normal (0) and Deviated (1) are the two states encoded by the binary target variable.. In directional drilling, trajectory departure is quantified by Dogleg Severity (DLS). To ensure engineering rigour, the ‘Deviated’ class is defined by an industry-standard geometric threshold exceeding 2.0° per 100 ft (0.0656°/m) [1,2], calculated from directional survey stations using the minimum curvature method. Trajectory intervals exceeding this threshold introduce heightened operational risks, including drill-string cyclic fatigue, casing wear, and differential sticking. Within the 112 deviated samples, 76 intervals (67.9%) represent moderate deviation (2.0°–4.0°/100 ft), while 36 intervals (32.1%) represent severe dogleg (>4.0°/100 ft). The labels were generated by depth-matching the LWD well logs to the definitive directional survey using standard spline interpolation. Crucially, this model detects existing deviation at the current bit depth, rather than predicting future trajectory changes. Given the operational priority of the minority (Deviated) class, the class distribution produces roughly 189 Normal and 112 Deviated samples, resulting in an imbalance and classified as a moderate imbalance, which still needs to be explicitly corrected.

**Table 2:** Well log features used in this classification framework.

No.	Feature	Log Type	Unit	Observed Range (Dataset)	Geomechanical Significance
1	Depth	Survey	m (TVD)	4000–4300	Represents overburden stress; deeper formations generally experience higher in-situ stress.
2	Gamma-ray	Radioactivity	API	28.29–80.65	Indicates shale content; higher GR corresponds to weaker, ductile formations.
3	Shale Volume (V_sh_)	Derived	Fraction	0.112–0.917	Higher V_sh_ reduces rock strength and stability.
4	Resistivity	Induction/Laterolog	Ω·m	0.15–1.02	Low resistivity indicates conductive fluids/fractures and weaker zones.
5	ΔT (Delta T)	Acoustic	μs/ft	110.53–143.56	Higher ΔT indicates lower elastic stiffness.
6	V_p_	Acoustic	ft/s	6966–9047	Higher V_p_ indicates stiffer, more competent rock.
7	V_s_	Acoustic	ft/s	2609–4426	Reflects shear rigidity and elastic behaviour.

The dataset used in this study consists of previously recorded well-log measurements. Therefore, all analyses were performed offline and do not represent live drilling operations. The proposed workflow is intended to demonstrate the feasibility of future real-time deployment after validation using streaming LWD data.

### 3.2 Preprocessing pipeline

A scikit-learn pipeline (or imbalanced-learn Pipeline when SMOTE is added) encompasses all preprocessing processes to ensure rigorous isolation between training and assessment data at every level. For every fold, the pipeline carries out the subsequent stages sequentially:

1. Median Imputation: Raw well log features occasionally contain missing measurements (encoded as null placeholders like −999.25). A SimpleImputer with a median strategy is applied as the first step in the pipeline to handle these gaps, using only training-fold statistics.
2. Standard Scaling: To guarantee that scale-sensitive algorithms (KNN, LR, MLP) function on a level feature space, features are normalised to zero mean and unit variance using training-fold statistics.
3. SMOTE Oversampling**:** Applied before classifier training and strictly following imputation and scaling. To address edge scenarios where the minority class is extremely small in each fold, the number of synthetic nearest neighbours is set to $\text{min}\left(\text{5},{\text{n}}_{\text{minority}}-\text{1}\right)$.

To prevent data leakage, all preprocessing steps—including standard scaling and SMOTE oversampling are now strictly isolated.

### 3.3 Class imbalance correction strategy

Two complementary techniques address the nearly 1.69:1 Normal-to-Deviated class ratio. First, sklearn estimators that natively accept a class weight parameter (LR, MLP, HGB) are configured with class weight = ‘balanced’, which scales individual sample loss contributions by the inverse class frequency. Second, SMOTE is integrated into the training pipeline for all five algorithms, generating synthetic Deviated samples from k-nearest minority neighbours exclusively within the training partition, preventing any synthetic information from contaminating the evaluation fold. This dual strategy is motivated by theoretical and empirical evidence that combined resampling and cost-sensitive learning outperforms either approach in isolation [25,27].To quantify the effect of this pipeline within the 10-fold cross-validation scheme, each training partition (comprising approximately 271 samples: ~ 170 Normal and ~101 Deviated) undergoes SMOTE oversampling. The algorithm generates approximately 69 synthetic minority samples per fold to establish an exact 1:1 class balance (~170 samples per class) before model fitting. Furthermore, sensitivity analysis was conducted on the choice of k-nearest neighbors (k∈{3,5,7}). The results indicated minimal variance in the macro-F1 score, and k=5 was subsequently selected as the optimal hyperparameter to maintain local interpolation fidelity without introducing excessive boundary smoothing.

### 3.4 Algorithms

**K-Nearest Neighbours (KNN):** A non-parametric, instance-based learner that classifies a sample by plurality vote among its k nearest training points in scaled feature space. Hyperparameters tuned: k ∈ {5,7,9,11,13,15}, distance metric ∈ {Euclidean, Manhattan}, and weighting scheme ∈ {uniform, distance}.

**Logistic Regression (LR):** A linear probabilistic classifier fitting a logistic sigmoid to the log-odds. The regularisation strength C ∈ {0.01, 0.1, 0.5, 2, 5, 10} and solver ∈ {lbfgs, liblinear} are optimised. L2 penalty is applied.

**Gaussian Naive Bayes (GNB):** A generative probabilistic classifier that assumes conditional feature independence given the class label. The variance smoothing parameter (var_smoothing ∈ [1 × 10^−10^, 1 × 10^−5^]) is tuned.

**Multilayer Perceptron (MLP):** A feedforward neural network with one or two hidden layers. Architecture ∈ {(32,16), (64,32), (128,64)}, activation ∈ {ReLU, tanh}, weight decay α∈ {0.0001, 0.001, 0.01}are searched.

**Histogram Gradient Boosting (HGB):** Scikit-learn’s native HistGradientBoostingClassifier -a histogram-binned implementation of gradient-boosted decision trees. Hyperparameters: max iter ∈ {150,250}, learning_rate ∈ {0.03,0.07,0.15}, max_depth ∈ {4,6,8}, L2 regularisation ∈ {0.5,2}, and min_samples_leaf ∈ {15,25}.

To ensure full reproducibility and address computational and structural parameter configurations, the complete hyperparameter search space, total candidate configurations evaluated, scoring metrics, and optimal parameters determined via 5-fold depth-blocked GridSearchCV are detailed in Table 3. All models were optimized for macro-averaged F1-score. For small-sample petrophysical data (N=301), Histogram Gradient Boosting incorporates histogram binning, which discretizes continuous log measurements into integer-valued bins, bounding split evaluations and providing intrinsic regularization against local variance.

**Table 3:** Hyperparameter search space evaluated candidate counts, and optimal parameters.

Algorithm	Evaluated Hyperparameter Grid	Total Candidates	Optimal Selected Parameters
**KNN**	n_neighbors: {5, 7, 9, 11, 13, 15} weights: uniform,distance} metric: euclidean, manhattan}	24	n_neighbors = 5 weights = ‘distance’ metric = ‘euclidean’
**LR**	C: 0.01, 0.1, 0.5, 2.0, 5.0, 10.0} solver: lbfgs, liblinear} penalty: l2}	12	C = 5.0 solver = ‘lbfgs’ penalty = ‘l2’
**GNB**	var_smoothing: {$\text{1}\times {\text{1}\text{0}}^{-\text{1}\text{0}},\text{1}\times {\text{1}\text{0}}^{-\text{9}},\text{1}\times {\text{1}\text{0}}^{-\text{7}},\text{1}\times {\text{1}\text{0}}^{-\text{5}}$}	4	var_smoothing=$\text{1}\times {\text{1}\text{0}}^{-\text{1}\text{0}}$
**MLP**	hidden_layer_sizes: {(32, 16), (64, 32), (128, 64)} activation: {relu, tanh} alpha: {0.0001, 0.001, 0.01}	18	hidden_layer_sizes= (64, 32) activation = ‘relu’ alpha = 0.0001
**HGB**	max_iter: {150, 250} learning_rate: {0.03, 0.07, 0.15} max_depth: {4, 6, 8} l2_regularization: {0.5, 2.0} min_samples_leaf: {15, 25}	72	max_iter = 250 learning_rate = 0.07 max_depth = 6 l2_regularization = 0.5 min_samples_leaf = 15

### 3.5 Experimental protocol

The evaluation protocol is designed to maximise statistical reliability given the modest sample size (n = 301):

1. Hyperparameter Tuning: 5-fold depth-blocked GridSearchCV conducted exclusively on a contiguous 70% depth-partitioned training subset (210 samples). Shuffling was disabled to maintain spatial contiguous blocks and avoid autocorrelation leakage between tuning partitions.
2. Primary Evaluation:. The optimally tuned estimators were subsequently evaluated across the entire 301-sample depth interval using 10-fold contiguous depth-blocked cross-validation. The dataset was partitioned into 10 sequential, non-overlapping spatial blocks of approximately 30 m each (~30 samples per fold).
3. Multi-Seed Stability: The entire 10-fold depth-blocked cross-validation evaluation is repeated across five independent random seeds. Mean ± standard deviation and 95% confidence intervals are reported for all headline metrics. The 95% confidence intervals are calculated using Student’s t-distribution across the 10 cross-validation folds (degrees of freedom df=n−1=9) according to the formula: ${\text{CI}}_{\text{9}\text{5}}=t_{\text{0}.\text{9}\text{7}\text{5},\text{9}}\times \left(s/\sqrt{\text{1}\text{0}}\right)$, where s is the sample standard deviation.

Overall Accuracy, Macro-F1, AUC-ROC, MCC, and per-class (Deviated) Precision, Recall, and F1-score are among the performance indicators provided for each algorithm. The best-performing technique is used to quantify feature importance via permutation importance on the held-out test partition, with n_repeats = 40 to produce stable importance estimations.

### 3.6 Generative AI statement

During the preparation of this manuscript, the authors used Google Gemini to assist with language editing, terminology harmonisation, grammar correction, and structural formatting. The AI tool was not used to generate, modify, analyse, or interpret the study data, machine-learning analyses, statistical results, figures, tables, or scientific conclusions. All AI-assisted outputs were critically reviewed, verified, and edited by the authors against the original manuscript, underlying analyses, references, and study records. The authors take full responsibility for the accuracy, validity, originality, and integrity of the final manuscript. The use of AI affected only the manuscript text related to language, terminology, and formatting, and did not affect the study data, analytical results, or supporting files.

## 4. Results

### 4.1 Exploratory data analysis

The exploratory data analysis (EDA) panel with six sub-plots is shown in Fig 1. The nearly 1.69:1 class imbalance is confirmed by the class distribution histogram, Fig 1(a), showing 189 normal samples (62.8%) and 112 deviated samples (37.2%). As predicted by elasticity theory, the feature correlation heat-map Fig 1(b) shows an exceptionally strong positive correlation between V_p_ and V_s_ (r = 0.99) and a near-perfect inverse correlation between Delta T and V_p_ (r = −0.99), consistent with their reciprocal mathematical relationship. The deterministic derivation of the V_sh_ log is confirmed by the perfect correlation between Shale Volume and Gamma-ray (r = 1.00). According to the Depth vs. Gamma-ray cross-plot Fig 1(c), deviated intervals are distributed across the entire 4,000–4,300 intervals rather than forming an isolated deep cluster. The use of non-linear classifiers is motivated by the complex, overlapping class boundaries revealed in the V_p_-V_s_ cross-plot Fig 1(e) and the shale volume distribution Fig 1(d). Furthermore, the resistivity–Delta T cross-plot Fig 1(f), colour-coded by depth, demonstrates that samples from shallower depths (darker purple shades) generally exhibit lower resistivity and lower elastic stiffness, which is consistent with porous or fluid-weakened formations.

**Fig 1:**
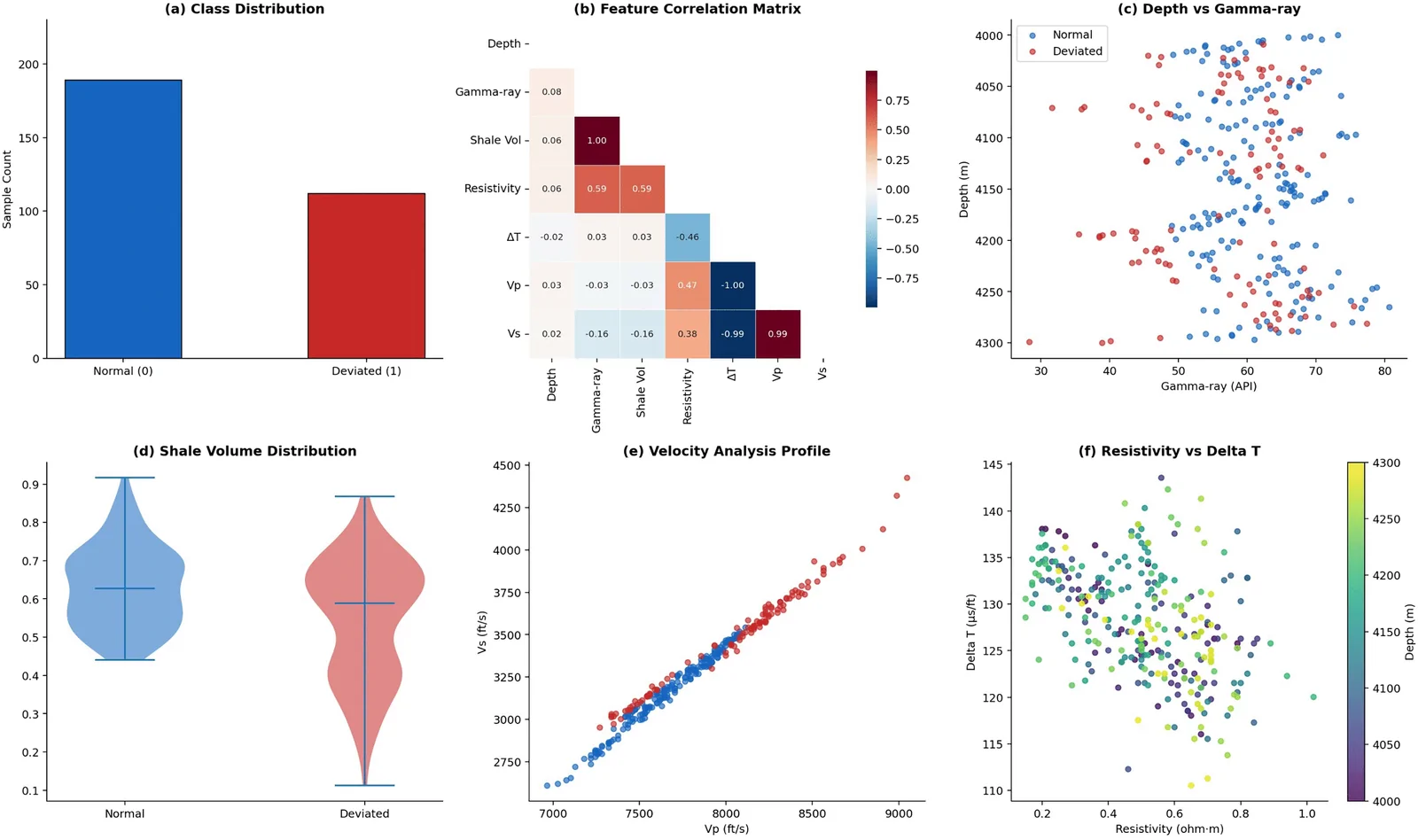
Exploratory data analysis: (a) class distribution, (b) feature correlation heat-map, (c) depth vs. classification, (d) shale volume distribution, (e) V_p_–V_s_ cross plot, (f) resistivity– Delta T cross plot.

### 4.2 Model performance-primary evaluation (depth block cv)

Table 4 reports the mean performance metrics obtained from the complete depth block cross-validation procedure. For visualisation purposes, Figs 2 and 3 present the confusion matrices and ROC curves obtained from a representative held-out test split using the optimal hyperparameters. These figures are intended to illustrate classifier behaviour, whereas the quantitative performance discussed in this study is based on the complete cross-validation results presented in Table 4. To isolate and validate the individual contributions of resampling and cost-sensitive penalties, an ablation study was conducted on the top-performing model (HGB). As detailed in Table 5, both SMOTE oversampling alone and the combined application of SMOTE with balanced class weights yielded the most robust performance (97.88% Macro-F1), substantially outperforming the unweighted baseline (96.85%) and the class-weights-only approach (97.19%)

**Table 4:** Mean performance metrics (mean ± 95% confidence interval) obtained from the complete depth block cross-validation across five random seeds.

Algorithm	Accuracy (%)	Macro-F1 (%)	AUC-ROC (%)	MCC	CV-MacroF1 CI95 (±%)
KNN	94.67	94.39	99.54	0.8929	4.17
LR	79.08	77.03	88.45	0.5900	7.67
GNB	83.10	81.04	93.87	0.6582	6.98
MLP	88.06	86.04	96.06	0.7489	6.36
HGB	98.00	97.88	98.92	0.9591	2.05

**Table 5:** Ablation analysis of class imbalance mitigation strategies for the top-performing model (HGB).

Imbalance Treatment Strategy	Macro-F1 (%)
Baseline (Unweighted, No Resampling)	96.85
Class Weights Alone (‘balanced’)	97.19
SMOTE Oversampling Alone (k=5)	97.88
SMOTE + Balanced Class Weights (Proposed)	97.88

**Fig 2:**
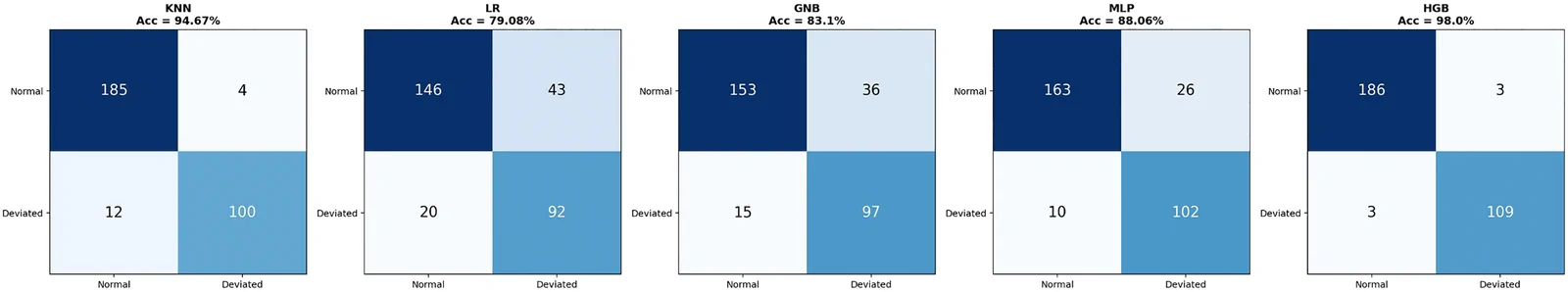
Representative confusion matrices obtained from a single held-out test fold during the 10-fold cross-validation using the optimal hyperparameters.

**Fig 3:**
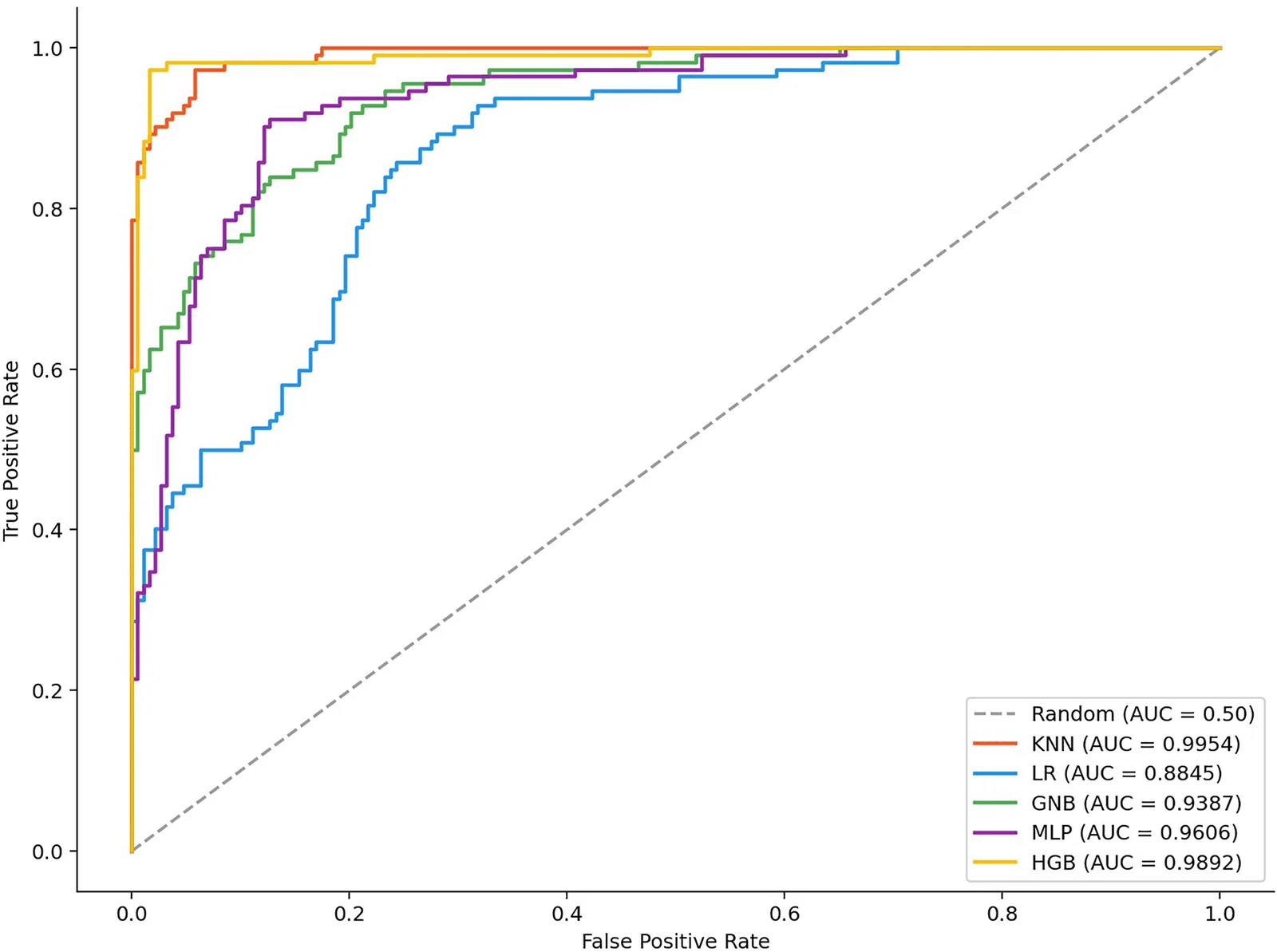
Representative ROC curves obtained from a single held-out test fold during the 10-fold cross-validation.

### 4.3 Feature importance analysis

Permutation-based feature significance scores for HGB, the top-performing algorithm, are shown in Fig 4. They were calculated on the held-out test partition using 40 random permutation repeats to get consistent estimates. The most significant characteristic is Delta T, which has a mean accuracy decline that is significantly greater than that of any other variable. In line with the geomechanical interpretation that argillaceous intervals are a primary mechanical driver, Gamma Ray ranks as the second most critical feature. In contrast, other features, including Vs, Vp, Resistivity, Shale Volume, and Depth, contribute minor or secondary classification signals in this dataset. However, it is important to acknowledge that permutation importance can be influenced by highly correlated predictors. For example, the strong multicollinearity between $V_{p}$ and $V_{s}$ (r=0.99), ΔT and $V_{p}$ (r=−0.99), and Gamma-ray and Shale Volume (r=1.00) can cause importance values to split among redundant features, potentially undervaluing their collective physical contribution. Therefore, these rankings represent model-based statistical associations rather than definitive physical causality.

**Fig 4:**
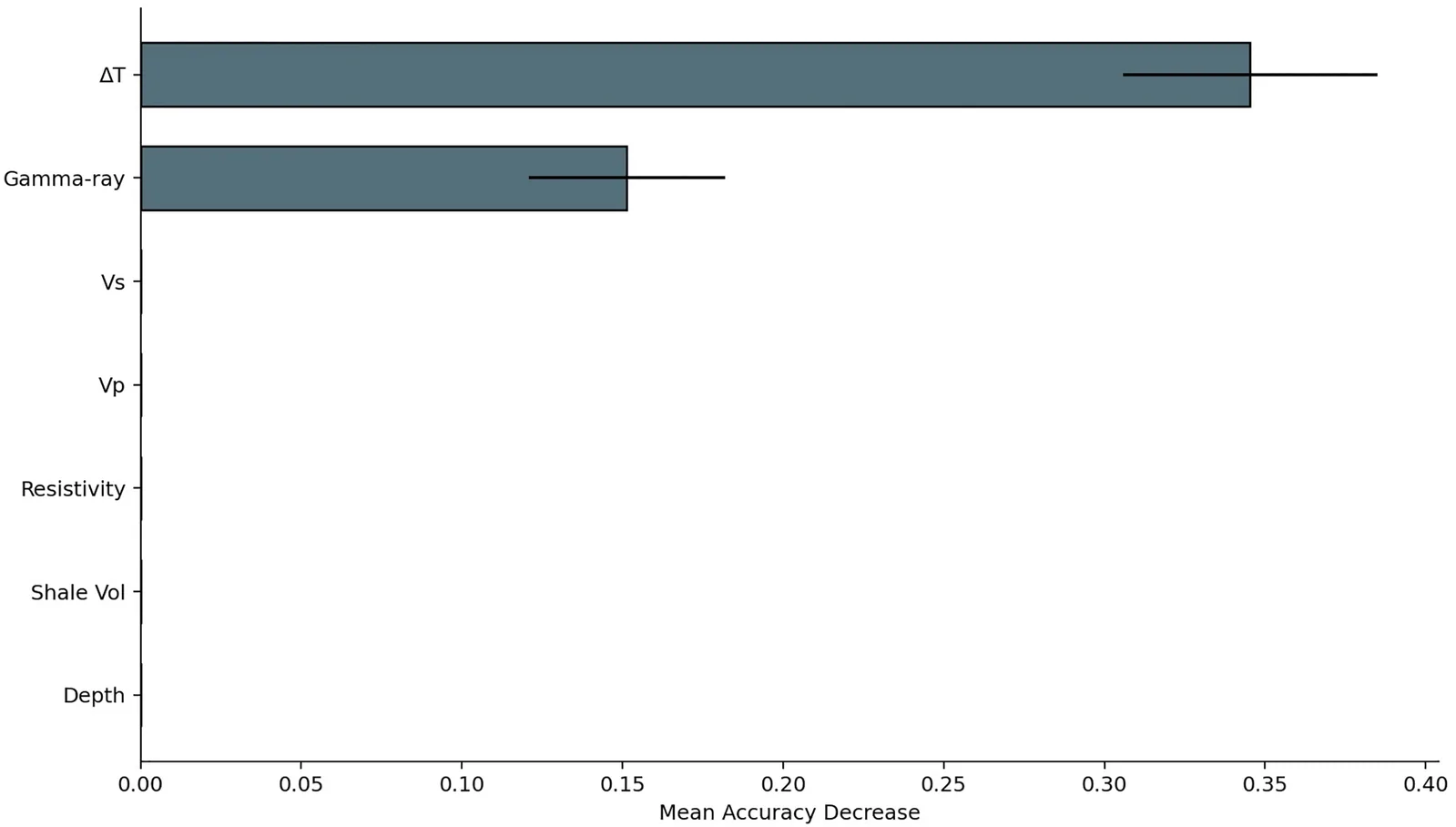
Permutation feature importance for HGB.

The learning curves for the optimal model and the depth block CV macro-F1 scores with 95% confidence intervals for all assessed classifiers are shown in Fig 5. As the sample size grows, the HGB learning curve shows a diminishing gap between training and validation accuracy, as seen in Fig 5(a). This indicates controlled overfitting and high generalisation features. With the complete training set, the validation accuracy plateaus at roughly 98.57%, while the training accuracy converges to 100%. The validation curve’s flattening indicates that the model has reached saturation, and more training data would probably result in minor performance gains. Fig 5(b) reflects this excellent performance, with HGB achieving the highest overall cross-validation score at 97.88% (±2.05%).

**Fig 5:**
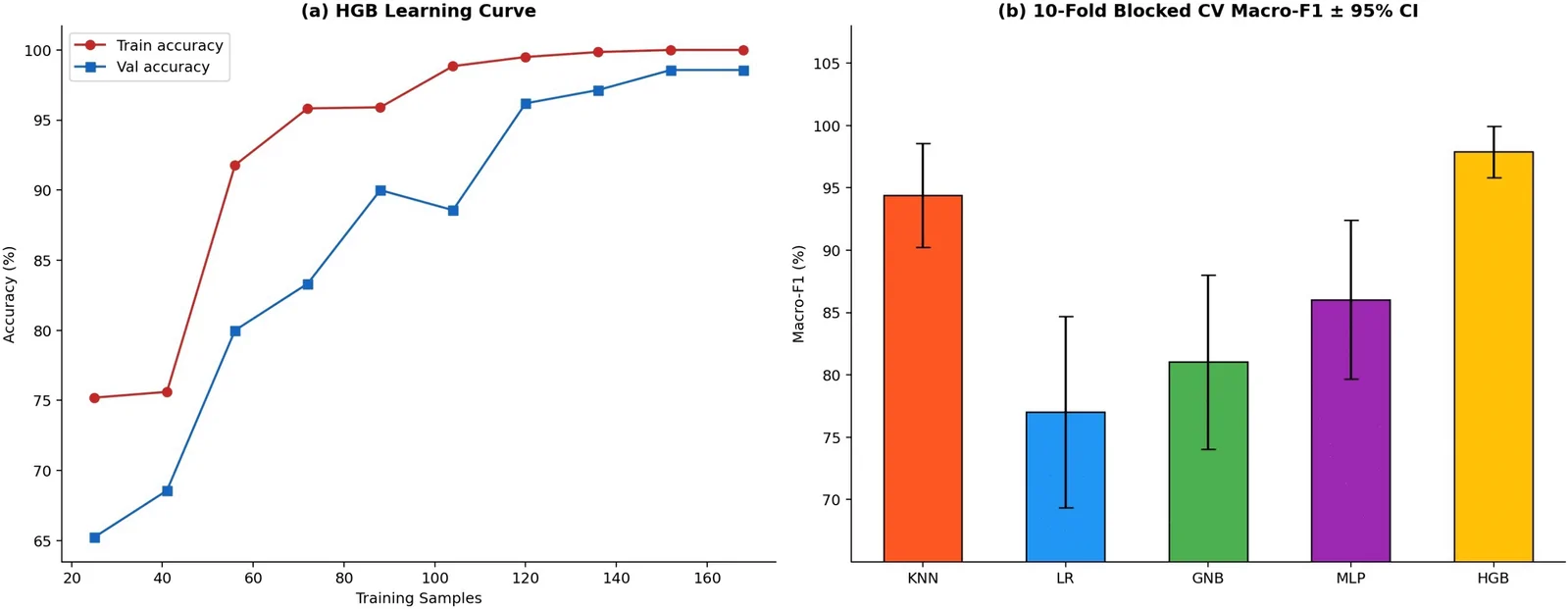
(a) Learning curves for the best model (HGB). (b) depth block CV macro-F1 scores with 95% CI error bars for all five algorithms.

To transition from qualitative feature importance to rigorous quantification, Partial Dependence Plots (PDP) were generated for the top two predictive features in Fig 6. The marginal response curves quantify the non-linear relationship between petrophysical properties and wellbore trajectory departure probability. Specifically, the PDP for ΔT in Fig 6(a) reveals a critical threshold effect, where the probability of borehole deviation rises sharply when acoustic slowness exceeds approximately 123 μs/ft. Similarly, the Gamma-ray PDP in Fig 6(b) demonstrates a steep risk transition at an API value of approximately 49, confirming that intervals characterised by higher acoustic slowness and elevated radioactivity exhibit a sharply increased susceptibility to trajectory departure.

**Fig 6:**
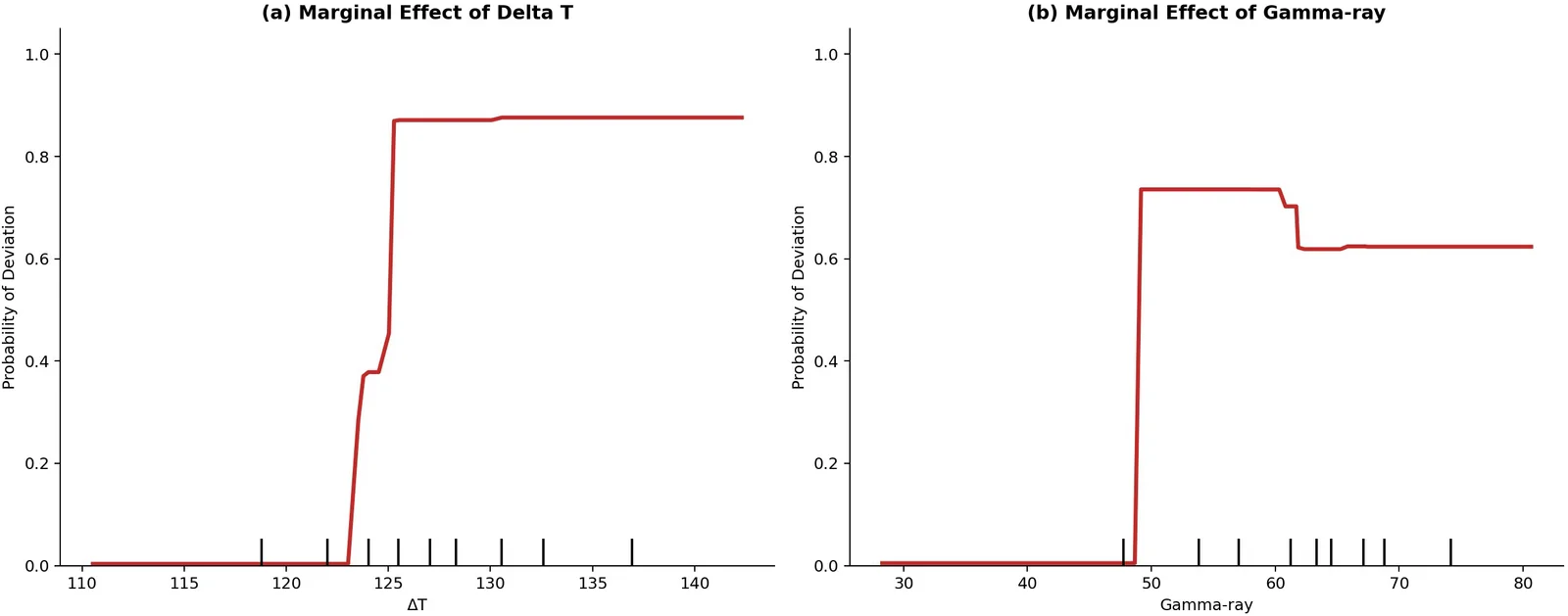
Partial dependence plots of top geomechanical features on deviation risk. (a) Marginal effect of ΔT and (b) Marginal effect of Gamma-ray.

### 4.4 Multi-seed stability analysis

The multi-seed stability findings for five separate random seeds are shown in Fig 7. In all four provided measures (Accuracy, Macro-F1, AUC-ROC, and Deviated-class F1), HGB has the highest mean values and the lowest standard deviations, indicating that its higher performance is reliable and not the result of a favourable random partition. MLP, on the other hand, exhibits the greatest variance among seeds, particularly in the Deviated-class F1 metric, indicating seed-sensitive behaviour that is likely caused by the stochastic nature of neural network weight initialisation and optimisation on small partitions. It’s interesting to note that, despite GNB’s intermediate performance, its remarkably tight error bars show that, independent of the data partition, its conditional independence assumption produces extremely stable, repeatable results.

**Fig 7:**
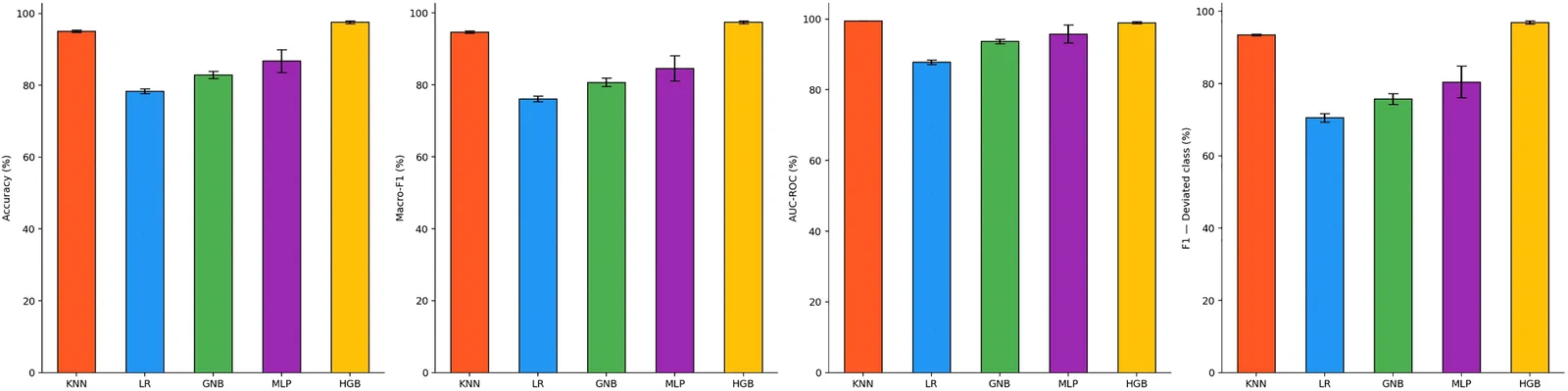
Multi-seed stability analysis across five random seeds.

To rigorously address generalisation and potential overfitting indicated by the remaining training-validation gap at maximum sample size in Fig 5(a), supplementary diagnostics were evaluated. Multi-seed stability testing across five independent random seeds in Fig 7 confirms minimal variance in macro-F1 and accuracy, indicating the gap is stable. Furthermore, an out-of-fold calibration plot (Reliability Diagram) was generated in Fig 8 to assess the alignment between predicted probabilities and actual deviation frequencies. As expected for tree-based models trained on balanced data, the HGB model exhibits a steep, sigmoidal calibration curve [25]. This distortion reflects the probability shift introduced by SMOTE oversampling and balanced class weighting, which pushes raw probabilities toward extremes [31]. However, despite this expected miscalibration, the model maintains exceptional structural discriminative capacity (AUC-ROC = 98.92%). This diagnostic confirms that while the raw probabilities require scaling for absolute risk interpretation, the model effectively separates the classes, and the minor learning curve gap represents irreducible lithological noise rather than standard overfitting.

**Fig 8:**
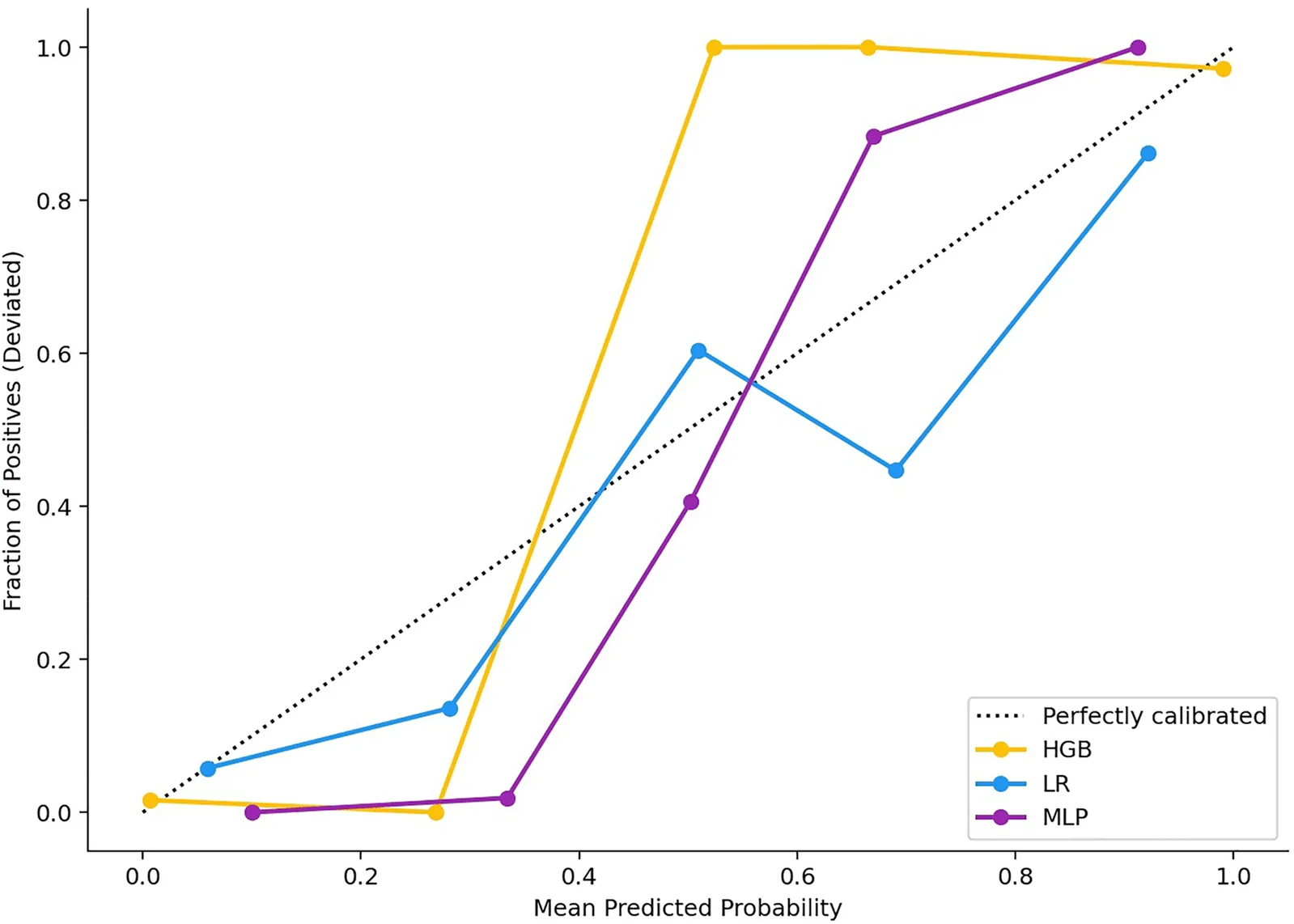
Calibration curves (reliability diagrams) for HGB, LR, and MLP classifiers.

## 5. Discussion

### 5.1 Algorithm and performance rationale

HGB consistently outperforms all five random seeds and all assessed metrics due to several inherent algorithmic benefits that are especially well-suited to the features of good log datasets. Through a step-by-step additive process, Histogram Gradient Boosting builds an ensemble of shallow decision trees, each of which corrects the residual mistakes of its predecessors [32].. This sequential error-correction mechanism effectively captures the intricate, non-linear interactions between petrophysical characteristics that control deviation risk. These interactions are not modelled by linear classifiers like LR and are assumed away by probabilistic classifiers like GNB through the conditional independence approximation. Here are two further benefits that are pertinent to this application when histogram binning is used instead of an accurate split-point search. First, it reduces overfitting on the small 301-sample dataset by discretising the continuous feature space, which provides implicit regularisation. Second, it can handle missing values natively, which is a useful feature considering that measurement gaps are frequently present in well log data. Strong recall on the minority Deviated class is achieved by sklearn’s HGB implementation, which provides native support for class weight and SMOTE oversampling in the training pipeline. This is crucial from an operational standpoint because a false negative (predicting Normal when the well is deviating) is far more expensive than a false positive.

Although MLP, which came in second overall, has the advantage of being able to learn multi-layer non-linear representations, its training is more susceptible to hyperparameter setup and initialisation, as evidenced by a marginally greater metric variance among seeds. Although LR and KNN offer helpful interpretable and instance-based baselines, respectively, performance on non-linearly separable deviation patterns is limited by their structural constraints. The significant pairwise correlations shown in the log feature space cause GNB to violate its Gaussian conditional independence assumption, despite being computationally simple to train (Fig 1, EDA correlation panel).

### 5.2 Feature importance and geomechanical interpretation

Fig 4 permutation relevance ranking is in good agreement with current geomechanical knowledge. The high ranking of Delta T and Gamma-ray supports established statistical associations between these petrophysical signatures and mechanical rock behaviour. Under drilling-induced stress perturbations, argillaceous formations, which are characterised by high clay mineral content and correspondingly high GR responses, exhibit time-dependent creep deformation. This process can gradually change the effective force balance on the drill string and cause bit walk [18,24]These quantitative thresholds align with established geomechanical principles. Higher ΔT values reflect lower formation elastic stiffness, while elevated Gamma-ray values indicate higher argillaceous (shale) content. The abrupt probability increases observed at 123 μs/ft and 49 API mark the critical material boundaries where formations transition into more ductile, clay-rich lithologies prone to time-dependent creep under in-situ stress, directly driving the observed wellbore deviations.

While contributing a secondary classification signal, Resistivity can be mechanistically interpreted through its inverse relationship with porosity and fluid saturation. Zones of abnormally low resistivity may indicate over-pressured or fractured intervals where pore pressure support is insufficient to maintain borehole wall integrity, resulting in localised wall failure that can deflect the bit [23].

### 5.3 Comparison with the literature baseline

Benchmarking the current study’s best model against directly comparable literature values reveals that HGB’s predictive metrics are competitive with the optimised Random Forest model presented by Bhattacherjee et al. [16] which achieved an exceptional testing accuracy of 96.67% and a robust AUC-ROC of 0.9975. The present study’s advantage lies in structural and methodological rigour. Specifically, our implementation of multi-seed stability analysis, strict SMOTE data-leakage prevention, and explicit 95% CI reporting provides a more resilient framework against overfitting compared to standard historical data splits. Furthermore, the use of Macro-F1 as our primary selection criterion, rather than overall accuracy, which can be artificially inflated by the majority class, makes the present evaluation highly conservative and operationally meaningful for wellbore quality assessment and trajectory risk classification [28].

### 5.4 Practical implications for drilling operations

The suggested framework’s deployability as a real-time decision-support tool is what makes it useful. The log curves utilised as features in this study can be transmitted to the surface in real time via mud-pulse or wired-pipe telemetry by LWD sensors installed above the drill bit. Geologists and reservoir engineers can use this automated framework to map formation-response risk zones in real-time, allowing for optimised casing design, wellbore stability modelling, and localised stress-state profiling. With just a serialised model artefact and a feature pre-processing module at the surface data aggregation node, integration with current wellsite automation and early kick detection systems is structurally simple.

### 5.5 Limitations and future work

The current study has several limitations that should be discussed. First, the analysis is based on a single-well dataset of 301 samples, which restricts generalisation to wells with other lithological sequences, tectonic settings, or drilling parameters, even though it is sufficient for the employed ML approaches given the depth block CV evaluation strategy. The next logical stages are transfer-learning techniques to apply the model to new fields or cross-well validation using offset well data from the same field. Second, a multi-class formulation that incorporates severity categories (such as mild, moderate, and severe deviation) or a regression-based method predicting continuous dogleg severity (DLS) may produce more useful results. The binary classification target (Normal vs. Deviated) collapses potentially informative gradations in deviation severity into a single boundary. Third, real-time drilling engineering parameters (WOB, RPM, flow rate, torque, drag) that are known to contribute to deviation are absent from the petrophysical feature set, despite its geomechanically motivation. It is anticipated that hybrid models, which incorporate both surface drilling mechanics parameters and subsurface geo-mechanical properties, will perform better than single-domain models and offer a viable avenue for future research.

Another limitation is that the proposed framework was evaluated using historical well-log data rather than live streaming LWD measurements. Consequently, the study demonstrates offline classification capability and does not validate real-time operational performance. Future work should evaluate computational latency, streaming data handling, and online model robustness during active drilling operations.

## 6. Conclusion

Seven Independent well log petrophysical parameters were used in this study to establish a rigorous, repeatable machine learning framework for binary borehole deviation categorisation. On a 301-sample LWD well log dataset derived from a single vertical well, five supervised classifiers were systematically compared under the same preprocessing, class-imbalance correction, hyperparameter optimisation, and multi-seed cross-validation conditions: KNN, Logistic Regression, Gaussian Naive Bayes, MLP Neural Network, and Histogram Gradient Boosting.

The Histogram Gradient Boosting classifier demonstrated statistically steady supremacy over the rival algorithms, consistently achieving the top performance across all assessment criteria and all five random seeds. In line with accepted geo-mechanical theory involving overburden stress, shale creep, and formation ductility as causative drivers of borehole trajectory deviation, feature importance analysis verified that Delta T, gamma-ray, and Resistivity are the main predictors of deviation risk.

This work’s methodological contributions are depth-block cross-validation with 95% CI reporting, dual-strategy class imbalance correction with leakage-safe SMOTE, and multi-seed stability analysis and improve the standard of evidence commonly found in the drilling ML literature and offer a repeatable evaluation template for upcoming comparative studies. With the potential to lower NPT and enhance wellbore quality in exploration and production drilling operations, this formation-response framework, although limited to a single well and lacking mechanical drilling controls (e.g., WOB, BHA design), serves as a proof-of-concept.

### Nomenclature

**Table:** 

Symbol/ Term	Description	Unit
TVD	True Vertical Depth	m
GR	Gamma-ray log	API
V_sh_	Shale Volume	V/V
Rt	True Formation Resistivity	Ω·m
ΔT	Compressional Slowness (Sonic Transit Time)	μs/ft
V_p_	Compressional Wave Velocity	ft/s
V_s_	Shear Wave Velocity	ft/s
RHOB	Bulk Density	g/cm³
Ρ	Density	g/cm³
DPHI	Density Porosity	V/V
Ν	Poisson’s Ratio	Dimensionless
y	Class label (Normal/Deviated)	–
X	Feature vector	–
N	Number of samples	–
TP	True Positives	–
TN	True Negatives	–
FP	False Positives	–
FN	False Negatives	–

## Supporting information

S1 File Machine learning Python notebook. The Jupyter Notebook (S1_File.pdf) containing the complete preprocessing, hyperparameter tuning, cross-validation, and visualization code used to generate the results in this study.
(PDF)

S2 File Machine learning Python notebook source code. The Jupyter Notebook (S2_File.ipynb) containing the executable code.
(IPYNB)

## Abbreviations

AI: Artificial Intelligence
API: American Petroleum Institute
ANN: Artificial Neural Network
AUC: Area Under the Receiver Operating Characteristic Curve
AUC-ROC: Area Under the Receiver Operating Characteristic Curve
CI: Confidence Interval
CV: Cross-Validation
EDA: Exploratory Data Analysis
F1: F1-score
GNB: Gaussian Naïve Bayes
HGB: Histogram Gradient Boosting
KNN: K-Nearest Neighbor
LR: Logistic Regression
MCC: Matthews Correlation Coefficient
ML: Machine Learning
MLP: Multi-Layer Perceptron
QC: Quality Control
RF: Random Forest
ROC: Receiver Operating Characteristic
SMOTE: Synthetic Minority Over-sampling Technique
SVM: Support Vector Machine
WOB: Weight on Bit
RPM: Revolutions Per Minute
ROP: Rate of Penetration
BHA: Bottom Hole Assembly
