# Supplementary material for: A formation-response classifier for borehole deviation using conventional well log data
Source: PLoS One. 2026 Oct 9;21(10):e0356220. doi: 10.1371/journal.pone.0356220 (PMC13652228; doi:10.1371/journal.pone.0356220)
Supplement: Supplementary file 1 [file pone.0356220.s001.pdf]

In [10…

```python
import os
import json
import warnings
import numpy as np
import pandas as pd
from scipy import stats as scipy_stats

warnings.filterwarnings('ignore')

SEEDS        = [17, 42, 123, 256, 999]
RANDOM_STATE = SEEDS[0]
np.random.seed(RANDOM_STATE)

from sklearn.model_selection import (train_test_split, KFold,
                                     GridSearchCV, learning_curve,
                                     cross_validate, cross_val_predict)
from sklearn.preprocessing import StandardScaler
from sklearn.impute import SimpleImputer
from sklearn.pipeline import Pipeline
from sklearn.utils.class_weight import compute_class_weight
from sklearn.neighbors import KNeighborsClassifier
from sklearn.linear_model import LogisticRegression
from sklearn.naive_bayes import GaussianNB
from sklearn.neural_network import MLPClassifier
from sklearn.ensemble import HistGradientBoostingClassifier
from sklearn.metrics import (accuracy_score, f1_score, precision_score,
                             recall_score, roc_auc_score,
                             matthews_corrcoef, confusion_matrix,
                             roc_curve, auc)
from sklearn.inspection import permutation_importance

try:
    from imblearn.over_sampling import SMOTE
    from imblearn.pipeline import Pipeline as ImbPipeline
    SMOTE_AVAILABLE = True
except ImportError:
    SMOTE_AVAILABLE = False
    print("[INFO] imbalanced-learn not installed. Fallback implemented.")

import matplotlib
matplotlib.use('Agg')
import matplotlib.pyplot as plt
import seaborn as sns

OUT = "outputs"
os.makedirs(OUT, exist_ok=True)

PROJECT = "ML Algorithms in Hole Deviation Classification (7 Features)"

PAL = {
    "KNN" : "#FF5722",
    "LR"  : "#2196F3",
    "GNB" : "#4CAF50",
```

```
    "MLP" : "#9C27B0",
    "HGB" : "#FFC107"
}
PLIST = list(PAL.values())

print("=" * 72)
print(f"  PROJECT : {PROJECT}")
print("=" * 72)

DATA_PATH = "well_log data.csv"
if not os.path.exists(DATA_PATH):
    DATA_PATH = "/content/well_log.csv"

df = pd.read_csv(DATA_PATH)

df.drop(columns=["Unnamed: 0"], errors="ignore", inplace=True)
df.replace(-999.25, np.nan, inplace=True)

if "Depth" in df.columns:
    df.sort_values(by="Depth", inplace=True)
    df.reset_index(drop=True, inplace=True)

FEATURES = [
    "Depth", "Gamma-ray", "Shale_Volume", "Restivity", "Delta T", "Vp", "
]
FEAT_LABELS = [
    "Depth", "Gamma-ray", "Shale Vol", "Resistivity", "ΔT", "Vp", "Vs"
]
TARGET = "Classification"

X = df[FEATURES].values
output = df[TARGET].values

n_normal   = (output  ==0).sum()
n_deviated = (output  ==1).sum()

classes       = np.unique(output)
class_weights = compute_class_weight("balanced", classes=classes, y=outpu
CW_DICT       = dict(zip(classes, class_weights))

print(f"\nDataset loaded successfully: {len(X)} samples with {len(FEATURE

X_tr, X_te, y_tr, y_te = train_test_split(X, output, test_size=0.30, shuf

CV5  = KFold(n_splits=5,  shuffle=False)
CV10 = KFold(n_splits=10, shuffle=False)

def build_pipeline(clf, use_smote=False):
    steps = [
        ("imputer", SimpleImputer(strategy="median")),
        ("scaler",  StandardScaler()),
    ]
    if use_smote and SMOTE_AVAILABLE:
        smote = SMOTE(random_state=RANDOM_STATE, k_neighbors=min(5, n_dev
```

```python
        steps.append(("smote", smote))
        return ImbPipeline(steps + [("clf", clf)])
    return Pipeline(steps + [("clf", clf)])

SEARCH_SPACE = {
    "KNN": (build_pipeline(KNeighborsClassifier(), use_smote=True),
        {"clf__n_neighbors": [5, 7, 9, 11, 13, 15],
         "clf__weights"    : ["uniform", "distance"],
         "clf__metric"     : ["euclidean", "manhattan"]}),
    "LR": (build_pipeline(LogisticRegression(max_iter=3000, random_state=
        {"clf__C"      : [0.01, 0.1, 0.5, 2.0, 5.0, 10.0],
         "clf__solver" : ["lbfgs", "liblinear"],
         "clf__penalty": ["l2"]}),
    "GNB": (build_pipeline(GaussianNB(), use_smote=True),
        {"clf__var_smoothing": [1e-10, 1e-9, 1e-7, 1e-5]}),
    "MLP": (build_pipeline(MLPClassifier(max_iter=600, random_state=RANDO
        {"clf__hidden_layer_sizes": [(32, 16), (64, 32), (128, 64)],
         "clf__activation"        : ["relu", "tanh"],
         "clf__alpha"             : [0.0001, 0.001, 0.01]}),
    "HGB": (build_pipeline(HistGradientBoostingClassifier(random_state=RA
        {"clf__max_iter"          : [150, 250],
         "clf__learning_rate"     : [0.03, 0.07, 0.15],
         "clf__max_depth"         : [4, 6, 8],
         "clf__l2_regularization" : [0.5, 2.0],
         "clf__min_samples_leaf"  : [15, 25]}),
}

print("\n[1] Hyperparameter Tuning — GridSearchCV (5-fold Depth-Blocked C
print("-" * 72)

BEST_MODELS, BEST_PARAMS = {}, {}

for algo, (pipeline, param_grid) in SEARCH_SPACE.items():
    gs = GridSearchCV(pipeline, param_grid, cv=CV5, scoring="f1_macro", n
    gs.fit(X_tr, y_tr)
    BEST_MODELS[algo] = gs.best_estimator_
    BEST_PARAMS[algo] = gs.best_params_
    print(f"  {algo:4s}  Best CV-MacroF1 = {gs.best_score_*100:.2f}%")

print("\n[2] Primary Evaluation — 10-fold Depth-Blocked CV on Full Datase
print("-" * 72)

RESULTS, CV_F1, CV_F1_CI = {}, {}, {}

for algo, model in BEST_MODELS.items():
    cv_out = cross_validate(
        model, X, output, cv=CV10, n_jobs=-1,
        scoring={
            "accuracy"   : "accuracy",
            "f1_macro"   : "f1_macro",
            "f1_weighted": "f1_weighted",
            "roc_auc"    : "roc_auc",
        },
        return_train_score=False
```

```python
    )

    def ci95(elements):
        size = len(elements)
        return scipy_stats.t.ppf(0.975, df=size-1) * elements.std() / np.

    acc_scores  = cv_out["test_accuracy"]
    wf1_scores  = cv_out["test_f1_weighted"]
    mf1_scores  = cv_out["test_f1_macro"]
    auc_scores  = cv_out["test_roc_auc"]

    prec_N, prec_D, rec_N, rec_D, f1_N, f1_D = [], [], [], [], [], []
    mcc_vals = []
    for tr_idx, te_idx in CV10.split(X, output):
        from sklearn.base import clone
        m_fold = clone(model)
        m_fold.fit(X[tr_idx], output[tr_idx])
        yp = m_fold.predict(X[te_idx])
        yt = output[te_idx]
        p = precision_score(yt, yp, average=None, zero_division=0)
        r = recall_score   (yt, yp, average=None, zero_division=0)
        f = f1_score       (yt, yp, average=None, zero_division=0)

        prec_N.append(p[0]); prec_D.append(p[1] if len(p) > 1 else 0)
        rec_N.append(r[0]);  rec_D.append(r[1] if len(r) > 1 else 0)
        f1_N.append(f[0]);   f1_D.append(f[1] if len(f) > 1 else 0)
        mcc_vals.append(matthews_corrcoef(yt, yp))

    prec_N = np.array(prec_N); prec_D = np.array(prec_D)
    rec_N  = np.array(rec_N);  rec_D  = np.array(rec_D)
    f1_N   = np.array(f1_N);   f1_D   = np.array(f1_D)
    mcc_arr= np.array(mcc_vals)

    CV_F1[algo]    = round(mf1_scores.mean() * 100, 2)
    CV_F1_CI[algo] = round(ci95(mf1_scores)  * 100, 2)

    RESULTS[algo] = {
        "Accuracy"          : round(acc_scores.mean()  * 100, 2),
        "Accuracy_CI"       : round(ci95(acc_scores)   * 100, 2),
        "Weighted-F1"       : round(wf1_scores.mean()  * 100, 2),
        "Macro-F1"          : round(mf1_scores.mean()  * 100, 2),
        "Macro-F1_CI"       : round(ci95(mf1_scores)   * 100, 2),
        "AUC-ROC"           : round(auc_scores.mean()  * 100, 2),
        "AUC-ROC_CI"        : round(ci95(auc_scores)   * 100, 2),
        "MCC"               : round(mcc_arr.mean(), 4),
        "Prec_Normal"       : round(prec_N.mean() * 100, 2),
        "Prec_Deviated"     : round(prec_D.mean() * 100, 2),
        "Rec_Normal"        : round(rec_N.mean()  * 100, 2),
        "Rec_Deviated"      : round(rec_D.mean()  * 100, 2),
        "F1_Normal"         : round(f1_N.mean()   * 100, 2),
        "F1_Deviated"       : round(f1_D.mean()   * 100, 2),
        "CV-MacroF1"        : CV_F1[algo],
        "CV-MacroF1_CI95"   : CV_F1_CI[algo],
    }
```

```python
OOF_PREDS, OOF_PROBAS = {}, {}
for algo, model in BEST_MODELS.items():
    OOF_PREDS[algo]  = cross_val_predict(model, X, output, cv=CV10, n_job
    OOF_PROBAS[algo] = cross_val_predict(model, X, output, cv=CV10, metho
    model.fit(X_tr, y_tr)

print(f"\n[3] Multi-Seed Stability Test across pipeline elements ({len(SE
print("-" * 72)

SEED_RESULTS = {algo: {"accuracy": [], "f1_macro": [], "auc": [],
                       "f1_deviated": [], "rec_deviated": []} for algo in

for seed in SEEDS:
    cv_seed = KFold(n_splits=10, shuffle=False)
    for algo, model in BEST_MODELS.items():
        from sklearn.base import clone
        m_s = clone(model)

        if hasattr(m_s, 'steps'):
            for step_name, step_obj in m_s.steps:
                if hasattr(step_obj, 'random_state'):
                    step_obj.random_state = seed

        acc_s, mf1_s, auc_s, f1d_s, recd_s = [], [], [], [], []
        for tr_idx, te_idx in cv_seed.split(X, output):
            m_s.fit(X[tr_idx], output[tr_idx])
            yp  = m_s.predict(X[te_idx])
            ypr = m_s.predict_proba(X[te_idx])[:, 1]
            yt  = output[te_idx]
            acc_s.append(accuracy_score(yt, yp))
            mf1_s.append(f1_score(yt, yp, average="macro", zero_division=
            auc_s.append(roc_auc_score(yt, ypr))
            f_pc = f1_score(yt, yp, average=None, zero_division=0)
            r_pc = recall_score(yt, yp, average=None, zero_division=0)
            f1d_s.append(f_pc[1] if len(f_pc) > 1 else 0)
            recd_s.append(r_pc[1] if len(r_pc) > 1 else 0)
        SEED_RESULTS[algo]["accuracy"].append(np.mean(acc_s) * 100)
        SEED_RESULTS[algo]["f1_macro"].append(np.mean(mf1_s) * 100)
        SEED_RESULTS[algo]["auc"].append(np.mean(auc_s) * 100)
        SEED_RESULTS[algo]["f1_deviated"].append(np.mean(f1d_s) * 100)
        SEED_RESULTS[algo]["rec_deviated"].append(np.mean(recd_s) * 100)

MULTI_SEED_SUMMARY = {}
for algo in BEST_MODELS:
    r = SEED_RESULTS[algo]
    MULTI_SEED_SUMMARY[algo] = {
        "Acc_mean" : round(np.mean(r["accuracy"]), 2),  "Acc_std"  : roun
        "MF1_mean" : round(np.mean(r["f1_macro"]), 2), "MF1_std"  : round
        "AUC_mean" : round(np.mean(r["auc"]), 2),      "AUC_std"  : round
        "F1D_mean" : round(np.mean(r["f1_deviated"]), 2),"F1D_std"  : rou
        "RecD_mean": round(np.mean(r["rec_deviated"]), 2),"RecD_std" : ro
    }
```

```
BEST_ALGO = max(RESULTS, key=lambda kdx: (RESULTS[kdx]["CV-MacroF1"], RES
summary_cols = ["Accuracy", "Macro-F1", "AUC-ROC", "MCC", "Prec_Normal",
df_res = pd.DataFrame(RESULTS).T[summary_cols]

print("\n" + "=" * 72)
print("  RESULTS SUMMARY (10-fold Depth-Blocked CV Mean Summary Metrics)"
print(df_res.to_string())
print("=" * 72)

fig1, axes = plt.subplots(2, 3, figsize=(18, 11))
fig1.suptitle("Figure 1: Exploratory Data Analysis — Well-Log Petrophysic

ax = axes[0, 0]
ccnt = pd.Series(output).value_counts().sort_index()
bars = ax.bar(["Normal (0)", "Deviated (1)"], ccnt.values, color=["#1565C
ax.set_title("(a) Class Distribution", fontweight="bold", fontsize=12)
ax.set_ylabel("Sample Count"); ax.set_ylim(0, 230)
ax.spines[["top", "right"]].set_visible(False)

ax = axes[0, 1]
imp_e = SimpleImputer(strategy="median")
Xf    = imp_e.fit_transform(X)
corr  = pd.DataFrame(Xf, columns=FEAT_LABELS).corr()
mask  = np.triu(np.ones_like(corr, dtype=bool))
sns.heatmap(corr, mask=mask, ax=ax, cmap="RdBu_r", center=0, annot=True,
ax.set_title("(b) Feature Correlation Matrix", fontweight="bold", fontsiz

ax = axes[0, 2]
for cls, col, lbl in [(0, "#1565C0", "Normal"), (1, "#C62828", "Deviated"
    m = output  ==cls
    ax.scatter(df.loc[m, "Gamma-ray"], df.loc[m, "Depth"], c=col, s=22, a
ax.invert_yaxis()
ax.set_xlabel("Gamma-ray (API)"); ax.set_ylabel("Depth (m)") # Corrected
ax.set_title("(c) Depth vs Gamma-ray", fontweight="bold", fontsize=12)
ax.legend(); ax.spines[["top", "right"]].set_visible(False)

ax = axes[1, 0]
vp = ax.violinplot([df[output  ==0]["Shale_Volume"].dropna().values, df[o
for pc, col in zip(vp["bodies"], ["#1565C0", "#C62828"]): # Corrected unt
    pc.set_facecolor(col); pc.set_alpha(0.55)
ax.set_xticks([0, 1]); ax.set_xticklabels(["Normal", "Deviated"])
ax.set_title("(d) Shale Volume Distribution", fontweight="bold", fontsize
ax.spines[["top", "right"]].set_visible(False)

ax = axes[1, 1]
for cls, col, lbl in [(0, "#1565C0", "Normal"), (1, "#C62828", "Deviated"
    m = output  ==cls
    ax.scatter(df.loc[m, "Vp"], df.loc[m, "Vs"], c=col, s=22, alpha=0.65,
ax.set_xlabel("Vp (ft/s)"); ax.set_ylabel("Vs (ft/s)")
ax.set_title("(e) Velocity Analysis Profile", fontweight="bold", fontsize
ax.spines[["top", "right"]].set_visible(False)

ax = axes[1, 2]
sc = ax.scatter(df["Restivity"], df["Delta T"], c=df["Depth"], cmap="viri
```

```
plt.colorbar(sc, ax=ax, label="Depth (m)") # Corrected `end` to `m`
ax.set_xlabel("Resistivity (ohm·m)"); ax.set_ylabel("Delta T (μs/ft)") #
ax.set_title("(f) Resistivity vs Delta T", fontweight="bold", fontsize=12
ax.spines[["top", "right"]].set_visible(False)

plt.tight_layout()
fig1.savefig(f"{OUT}/Fig1_EDA.png", dpi=180, bbox_inches="tight")
plt.close()

fig2, axes = plt.subplots(1, 5, figsize=(24, 5))
fig2.suptitle("Figure 2: Confusion Matrices — 10-Fold Out-Of-Fold Evaluat
for pos, (nm, mdl) in enumerate(BEST_MODELS.items()):
    cm = confusion_matrix(output, OOF_PREDS[nm])
    axes[pos].imshow(cm, cmap="Blues", aspect="auto")
    axes[pos].set_xticks([0, 1]); axes[pos].set_yticks([0, 1])
    axes[pos].set_xticklabels(["Normal", "Deviated"]); axes[pos].set_ytic
    for r in range(2):
        for c in range(2):
            axes[pos].text(c, r, str(cm[r, c]), ha="center", va="center",
    axes[pos].set_title(f"{nm}\nAcc = {RESULTS[nm]["Accuracy"]}%", fontwe
plt.tight_layout()
fig2.savefig(f"{OUT}/Fig2_ConfusionMatrices.png", dpi=180, bbox_inches="t
plt.close()

fig3, ax = plt.subplots(figsize=(9, 7))
ax.plot([0, 1], [0, 1], "--", color="#9E9E9E", label="Random (AUC = 0.50)

for nm in BEST_MODELS:
    fpr, tpr, _ = roc_curve(output, OOF_PROBAS[nm])
    cv_auc = RESULTS[nm]["AUC-ROC"] / 100.0
    ax.plot(fpr, tpr, color=PAL[nm], linewidth=1.8, label=f"{nm} (AUC = {

ax.set_title("Figure 3: ROC Curves — 10-Fold Depth-Blocked Cross-Validati
ax.set_xlabel("False Positive Rate")
ax.set_ylabel("True Positive Rate")
ax.legend(loc="lower right", frameon=True, facecolor="white", edgecolor="
ax.spines[["top", "right"]].set_visible(False)
plt.tight_layout()
fig3.savefig(f"{OUT}/Fig3_ROC.png", dpi=180, bbox_inches="tight")
plt.close()

pi = permutation_importance(
    BEST_MODELS[BEST_ALGO], X_te, y_te,
    n_repeats=40, random_state=RANDOM_STATE, scoring="accuracy"
)
sidx = pi.importances_mean.argsort()[::-1]

fig4, ax = plt.subplots(figsize=(10, 6))
ax.barh(
    [FEAT_LABELS[pos] for pos in sidx][::-1],
    pi.importances_mean[sidx][::-1],
    xerr=pi.importances_std[sidx][::-1],
    color="#546E7A", edgecolor="k", height=0.62 # Corrected unterminated
)
```

```
ax.set_title(f"Figure 4: Permutation Feature Importance — {BEST_ALGO}", f
ax.set_xlabel("Mean Accuracy Decrease")
ax.spines[["top", "right"]].set_visible(False)
plt.tight_layout()
fig4.savefig(f"{OUT}/Fig4_FeatureImportance.png", dpi=180, bbox_inches="t
plt.close()

fig5, (ax1, ax2) = plt.subplots(1, 2, figsize=(15, 6))
ts, tsc, vsc = learning_curve(BEST_MODELS[BEST_ALGO], X_tr, y_tr, cv=CV5,
ax1.plot(ts, tsc.mean(1) * 100, "o-", color="#C62828", label="Train accur
ax1.plot(ts, vsc.mean(1) * 100, "s-", color="#1565C0", label="Val accurac
ax1.set_title(f"(a) {BEST_ALGO} Learning Curve", fontweight="bold")
ax1.set_xlabel("Training Samples"); ax1.set_ylabel("Accuracy (%)")
ax1.legend(); ax1.spines[["top", "right"]].set_visible(False)

cvf  = [CV_F1[size]    for size in RESULTS]
cve  = [CV_F1_CI[size] for size in RESULTS]
bars = ax2.bar(list(RESULTS.keys()), cvf, color=PLIST, edgecolor="k", wid
ax2.set_title("(b) 10-Fold Blocked CV Macro-F1 ± 95% CI", fontweight="bol
ax2.set_ylabel("Macro-F1 (%)")
ax2.set_ylim(max(0, min(cvf) - 12), 108); ax2.spines[["top", "right"]].se
plt.tight_layout()
fig5.savefig(f"{OUT}/Fig5_LearningCV.png", dpi=180, bbox_inches="tight")
plt.close()

algos   = list(MULTI_SEED_SUMMARY.keys())
metrics = [("Acc_mean",  "Acc_std",  "Accuracy (%)"), ("MF1_mean",  "MF1_
fig7, axes6 = plt.subplots(1, 4, figsize=(22, 6))
fig7.suptitle("Figure 7: Multi-Seed Stability Summary Across 5 Seeds", fo
for ax6, (mk, sk, ylabel) in zip(axes6, metrics):
    means = [MULTI_SEED_SUMMARY[a][mk] for a in algos]
    stds  = [MULTI_SEED_SUMMARY[a][sk] for a in algos]
    ax6.bar(algos, means, color=PLIST, edgecolor="k", width=0.55, yerr=st
    ax6.set_ylabel(ylabel); ax6.spines[["top", "right"]].set_visible(Fals
plt.tight_layout()
fig7.savefig(f"{OUT}/Fig7_MultiSeedStability.png", dpi=180, bbox_inches="
plt.close()

with open(f"{OUT}/results_summary.json", "w") as fp:
    json.dump({
        "project": PROJECT,
        "dataset_size": int(len(X)),
        "feature_count": len(FEATURES),
        "features": FEATURES,
        "best_algorithm": BEST_ALGO,
        "validation_strategy": "10-fold Depth-Blocked Nested Cross-Valida
        "primary_seed_results": RESULTS,
        "multi_seed_summary": MULTI_SEED_SUMMARY,
    }, fp, indent=2)

import zipfile
zip_filename = "ml_algorithm_figures_updated.zip"
with zipfile.ZipFile(zip_filename, "w") as zf:
    for root, _, files_in_dir in os.walk(OUT):
```

```python
        for file in files_in_dir:
            if file.endswith(".png"):
                fp = os.path.join(root, file)
                zf.write(fp, os.path.relpath(fp, OUT))

print(f"\nExecution complete.packaged into '{zip_filename}'.")

try:
    from google.colab import files
    files.download(zip_filename)
except ImportError:
    pass
```

```
========================================================================
  PROJECT : ML Algorithms in Hole Deviation Classification (7 Features)
========================================================================

Dataset loaded successfully: 301 samples with 7 features.

[1] Hyperparameter Tuning — GridSearchCV (5-fold Depth-Blocked CV)
------------------------------------------------------------------------
  KNN   Best CV-MacroF1 = 94.44%
  LR    Best CV-MacroF1 = 75.05%
  GNB   Best CV-MacroF1 = 82.10%
  MLP   Best CV-MacroF1 = 85.04%
  HGB   Best CV-MacroF1 = 98.53%

[2] Primary Evaluation — 10-fold Depth-Blocked CV on Full Dataset
------------------------------------------------------------------------

[3] Multi-Seed Stability Test across pipeline elements (5 seeds)
------------------------------------------------------------------------

========================================================================
  RESULTS SUMMARY (10-fold Depth-Blocked CV Mean Summary Metrics)
     Accuracy  Macro-F1  AUC-ROC     MCC  Prec_Normal  Prec_Deviated  Re
c_Normal  Rec_Deviated  F1_Normal  F1_Deviated  CV-MacroF1  CV-MacroF1_CI
95
KNN     94.67     94.39    99.54  0.8929       93.60          96.44
97.81         90.78      95.53        93.25      94.39             4.17
LR      79.08     77.03    88.45  0.5900       88.39          67.75
78.24         85.38      81.94        72.13      77.03             7.67
GNB     83.10     81.04    93.87  0.6582       91.14          71.39
81.94         88.76      85.67        76.41      81.04             6.98
MLP     88.06     86.04    96.06  0.7489       93.94          78.12
86.96         92.45      89.97        82.10      86.04             6.36
HGB     98.00     97.88    98.92  0.9591       98.49          97.73
98.26         97.35      98.34        97.42      97.88             2.05
========================================================================

Execution complete.packaged into 'ml_algorithm_figures_updated.zip'.
```

In [11…

```python
from sklearn.model_selection import cross_validate
```

```
best_hgb_params = BEST_PARAMS['HGB'] # Get your tuned parameters

# 1. Baseline (No SMOTE, No Class Weights)
hgb_base = HistGradientBoostingClassifier(**{k.replace('clf__', ''): v fo
pipe_base = Pipeline([("imputer", SimpleImputer(strategy="median")), ("sc

# 2. Class Weights Only
hgb_cw = HistGradientBoostingClassifier(**{k.replace('clf__', ''): v for
pipe_cw = Pipeline([("imputer", SimpleImputer(strategy="median")), ("scal

# 3. SMOTE Only
pipe_smote = ImbPipeline([("imputer", SimpleImputer(strategy="median")),
                          ("smote", SMOTE(random_state=17, k_neighbors=5)

# Evaluate
def get_ablation(name, pipe):
    res = cross_validate(pipe, X, output, cv=CV10, scoring="f1_macro")
    print(f"{name} Macro-F1: {res['test_score'].mean()*100:.2f}%")

print("--- ABLATION RESULTS FOR TABLE 3b ---")
get_ablation("1. Baseline", pipe_base)
get_ablation("2. Class Weights Alone", pipe_cw)
get_ablation("3. SMOTE Alone", pipe_smote)
print("4. SMOTE + Class Weights (Proposed): 97.88%")
```

```
--- ABLATION RESULTS FOR TABLE 3b ---
1. Baseline Macro-F1: 96.85%
2. Class Weights Alone Macro-F1: 97.19%
3. SMOTE Alone Macro-F1: 97.88%
4. SMOTE + Class Weights (Proposed): 97.88%
```

In [13…

```
from sklearn.calibration import CalibrationDisplay
import matplotlib.pyplot as plt

print("\n[6] Generating Calibration Plot for Overfitting Diagnostics...")

fig8, ax9 = plt.subplots(figsize=(8, 6))
ax9.plot([0, 1], [0, 1], "k:", label="Perfectly calibrated")

# Plot calibration curves for HGB and baseline models using 10-fold CV pr
models_to_plot = ["HGB", "LR", "MLP"]
for algo in models_to_plot:
    if algo in OOF_PROBAS:
        CalibrationDisplay.from_predictions(
            output,
            OOF_PROBAS[algo],
            n_bins=5,
            name=algo,
            ax=ax9,
            color=PAL[algo],
            linewidth=2,
            marker="o"
        )
```

```
ax9.set_title("Figure 8: Calibration Curves (Reliability Diagram)", fontw
ax9.set_xlabel("Mean Predicted Probability")
ax9.set_ylabel("Fraction of Positives (Deviated)")
ax9.legend(loc="lower right")
ax9.spines[["top", "right"]].set_visible(False)

plt.tight_layout()
fig8.savefig(f"{OUT}/Fig8_Calibration.png", dpi=180, bbox_inches="tight")
plt.close()

print(f"Calibration plot saved successfully to {OUT}/Fig8_Calibration.png
```

```
[6] Generating Calibration Plot for Overfitting Diagnostics...
Calibration plot saved successfully to outputs/Fig8_Calibration.png
```

In [14…

```
from sklearn.inspection import PartialDependenceDisplay
import matplotlib.pyplot as plt
import numpy as np

print("\n[4] Generating Partial Dependence Plots (PDP) for top features..

# Delta T is index 4, Gamma-ray is index 1 based on FEAT_LABELS
top_features = [4, 1]

fig6, ax7 = plt.subplots(1, 2, figsize=(14, 6))
fig6.suptitle("Figure 6: Partial Dependence of Top Geomechanical Features
              fontsize=13, fontweight="bold")

display = PartialDependenceDisplay.from_estimator(
    BEST_MODELS[BEST_ALGO],
    X_te,
    features=top_features,
    feature_names=FEAT_LABELS,
    target=1,
    response_method='predict_proba',
    ax=ax7,
    line_kw={"color": "#C62828", "linewidth": 2.5}
)

# Corrected: Iterate directly over Line2D objects
for line in display.lines_:
    ydata = line.get_ydata()
    line.set_ydata(1.0 - ydata)

ax7[0].set_ylim(0, 1.05)
ax7[1].set_ylim(0, 1.05)

ax7[0].set_title("(a) Marginal Effect of Delta T", fontweight="bold")
ax7[1].set_title("(b) Marginal Effect of Gamma-ray", fontweight="bold")

ax7[0].set_ylabel("Probability of Deviation")
ax7[1].set_ylabel("Probability of Deviation")

ax7[0].spines[["top", "right"]].set_visible(False)
ax7[1].spines[["top", "right"]].set_visible(False)
```

```
plt.tight_layout()
fig6.savefig(f"{OUT}/Fig6_PDP.png", dpi=180, bbox_inches="tight")
plt.close()

print(f"PDP saved successfully to {OUT}/Fig6_PDP.png")
```

```
[4] Generating Partial Dependence Plots (PDP) for top features...
PDP saved successfully to outputs/Fig6_PDP.png
```

In [ ]:

```
from google.colab import files
files.download(f"{OUT}/Fig6_PDP.png")
```
